# Longitudinal associations between parents’ motivations to exercise and their moderate-to-vigorous physical activity

**DOI:** 10.1016/j.psychsport.2019.04.007

**Published:** 2019-07

**Authors:** Lydia G. Emm-Collison, Russell Jago, Ruth Salway, Janice L. Thompson, Simon J. Sebire

**Affiliations:** a Centre for Exercise, Nutrition & Health Sciences, School for Policy Studies, University of Bristol, 8 Priory Road, Bristol, BS8 1TZ, UK; b School of Sport, Exercise and Rehabilitation Sciences, University of Birmingham, Birmingham, B15 2TT, UK

**Keywords:** Motivation, Physical activity, Longitudinal, Parents, Accelerometer

## Abstract

**Objectives:**

This study is the first examination of the longitudinal associations between behavioural regulation and accelerometer-assessed physical activity in parents of primary-school aged children.

**Design:**

A cohort design using data from the B-Proact1v project.

**Method:**

There were three measurement phases over five years. Exercise motivation was measured using the BREQ-2 and mean minutes of moderate-to-vigorous physical activity (MVPA) were derived from ActiGraph accelerometers worn for a minimum of 3 days. Cross-sectional associations were explored via linear regression models using parent data from the final two phases of the B-Proact1v cohort, when children were 8–9 years-old (925 parents, 72.3% mothers) and 10 to 11 years-old (891 parents, 72.6% mothers). Longitudinal associations across all three phases were explored using multi-level models on data from all parents who provided information on at least one occasion (2374 parents). All models were adjusted for gender, number of children, deprivation indices and school-based clustering.

**Results:**

Cross-sectionally, identified regulation was associated with 5.43 (95% CI [2.56, 8.32]) and 4.88 (95% CI [1.94, 7.83]) minutes more MVPA per day at times 2 and 3 respectively. In the longitudinal model, a one-unit increase in introjected regulation was associated with a decline in mean daily MVPA of 0.52 (95% CI [-0.88, −0.16]) minutes per year.

**Conclusions:**

Interventions to promote the internalisation of personally meaningful rationales for being active, whilst ensuring that feelings of guilt are not fostered, may offer promise for facilitating greater long-term physical activity engagement in parents of primary school age children.

## Highlights

• This paper presents the first data examining the longitudinal associations between behavioural regulations and physical activity in adults.
• Greater personal value of exercise was associated with higher accelerometer-estimated moderate-to-vigorous physical activity.
• Higher levels of guilt and shame related to not being physically activity were associated with a decrease in physical activity over time.

## 1 Introduction

Physical activity is associated with reduced risk of a variety of health outcomes, including heart disease, stroke, type 2 diabetes, several forms of cancer, and depression (Kyu et al., 2016; Rebar et al., 2015). Similarly, physical inactivity has been shown to be detrimental for health and well-being and has been identified as a source of great economic cost globally (Ding et al., 2016). As such, global physical activity guidelines recommend that adults undertake at least 150 min per week of moderate intensity activity, including additional muscle-strengthening activities at least twice a week, alongside the general aim of reducing their sedentary time (Australian Government, 2017; Department for Department of Health, 2011; Health Council of the Netherlands, 2017; Tremblay et al., 2017; World Health Organisation, 2010). However, evidence suggests that between 15% and 43% of adults in western countries do not meet physical activity recommendations (Colley et al., 2011; Craig, Mindell, & Hirani, 2011; Hallal et al., 2012; Kapteyn et al., 2018). From a public health perspective, it is clear that efforts need to be made to encourage more adults to be regularly active.

Thirty-five percent of adults (aged 18–65) in the UK have dependent children (Office for National Statistics, 2017). Parents of young children have been shown to engage in less moderate-to-vigorous physical activity (MVPA) than similar aged adults without children (Bellows-Riecken & Rhodes, 2008; Berge, Larson, Bauer, & Neumark-Sztainer, 2011), with a noticeable decrease in physical activity at the point of transition to parenthood (Hull et al., 2010; McIntyre & Rhodes, 2009). Engaging in regular physical activity may be particularly challenging for parents with dependent children due to increased demands on their time, financial burdens, and a change in priorities compared to before parenthood. Yet, promoting parental engagement in physical activity could be beneficial for both parents and children in terms of health benefits, parenting behaviour, and energy levels (Hamilton & White, 2010; Lewis & Ridge, 2005). Additionally, if parents are active then they model active behaviour to their children (Sebire et al., 2016), with some evidence suggesting a weak positive association between parent physical activity and child activity (Jago et al., 2017a, Jago et al., 2017b; Shutz, Browning, Smith, Lohse, & Cunningham-Sabo, 2018; Yao & Rhodes, 2015).

A recent review of reviews highlighted that individual level variables (e.g., motivation, age, and health intentions) are the most consistent correlates of physical activity (Choi, Lee, Lee, Kang, & Choi, 2017) therefore indicating that interventions should be either tailored to specific populations or should encompass ways of manipulating these variables to increase physical activity. Self-determination theory (SDT; Ryan & Deci, 2017) is a framework through which the motivational processes that underpin physical activity can be investigated. Within SDT, quality of motivation is placed upon a continuum whereby different types of motivation differ in the extent to which they are autonomous or controlled (Deci & Ryan, 2000; Howard, Gagne, & Bureau, 2017). Three types of motivation are said to be more autonomous in nature: Intrinsic motivation, the most autonomous form of motivation characterised by an individual inherently enjoying or gaining satisfaction from the activity; integrated regulation, when the behaviour aligns with an individual’s identity; and identified regulation, when an individual consciously values the behaviour (Ryan & Deci, 2017). More controlled types of motivation are introjected regulation, when behaviour is controlled by self-imposed sanctions, such as shame, pride, ego, or guilt (Deci & Ryan, 2002) and external regulation, the most controlled form of motivation when behaviour is driven by external factors such as rewards, compliance and punishments (Deci & Ryan, 1987). Additionally, a lack of either autonomous or controlled forms of motivation is classed as amotivation (Ryan & Deci, 2000). In the context of physical activity, effortful and persistent behaviour is more likely to occur when an individual’s motivation is autonomous as opposed to controlled (Standage & Ryan, 2012).

Cross-sectional evidence consistently shows autonomous motivation for exercise (i.e., intrinsic, integrated, and identified regulation) to be positively associated with self-reported and accelerometer-assessed physical activity in healthy adults (see Teixeira, Carraca, Markland, Silva, & Ryan, 2012 for a review). Controlled motivation is generally shown to have little cross-sectional association with self-reported and accelerometer-assessed physical activity behaviour (Teixeira et al., 2012). However, when analysed separately, introjected regulation more frequently shows a positive cross-sectional association with physical activity whereas external regulation is more commonly negatively associated with physical activity (e.g., Edmunds, Ntoumanis, & Duda, 2006; Wilson, Rodgers, & Fraser, 2002). In addition to the cross-sectional evidence, there are a small number of studies that have examined, and provided evidence, for a small to moderate positive association between autonomous motivation and self-reported physical activity over periods of time ranging from 1 to 6 months (Barbeau, Sweet, & Fortier, 2009; Fortier, Kowal, Lemyre, & Orpana, 2009; Gunnell, Crocker, Mack, Wilson, & Zumbo, 2014). In support of these longitudinal associations, qualitative evidence aligns with the theoretical tenet that movement through the behavioural regulation continuum towards more autonomous motivation is central to physical activity adherence (Kinnafick, Thogerson-Ntoumani, & Duda, 2014). To date, studies have shown no evidence for a longitudinal association between controlled motivation and physical activity (Barbeau et al., 2009; Gunnell et al., 2014). The limited number of studies assessing the associations between motivation and physical activity over time have used autonomous and controlled composites and not disaggregated the types of behavioural regulation in statistical models, thus limiting the study of the roles of qualitatively different types of motivation. Additionally, these longitudinal studies have also relied on self-reported measures of physical activity behaviour which are prone to bias (Sallis & Saelens, 2000). Therefore, further investigation of the longitudinal associations between behavioural regulation and physical activity, using more reliable behavioural estimates (e.g., through accelerometers) is warranted.

Despite evidence indicating lower levels of physical activity in parents compared to the wider adult population, theoretical models have been seldom used to understand physical activity during parenthood (Bellows-Riecken & Rhodes, 2008). The quality of motivation for exercise may be particularly pertinent to parents’ physical activity due to extensive competing demands (e.g., time, fatigue, childcare) that may make converting some forms of motivation to behaviour more challenging for parents than non-parents (McIntyre & Rhodes, 2009; Solomon-Moore et al., 2016.) In support of this, in a study involving 1067 parents of children aged 5–6 years old, only identified regulation showed evidence of a cross-sectional association with MVPA after adjustment (Solomon-Moore et al., 2016), suggesting that, for parents of younger children, identifying with personally meaningful and valuable benefits of exercise may be the strongest motivational driver. However, there are no studies of the motivation-physical activity associations amongst parents of older children or evidence for any longitudinal associations between motivation and physical activity behaviour during parenthood.

The aims of this research were to 1) examine the cross-sectional associations between the behavioural regulations set forward in SDT and objectively-estimated physical activity in parents of children aged 8–9 years old and then two years later when the same child was 10–11 years old and 2) assess the longitudinal associations between behavioural regulation type and accelerometer-assessed physical activity in parents over a five-year period.

## 2 Method

### 2.1 Participants and procedure

The current analyses used data from the B-Proact1v project. The broader project is a longitudinal study exploring the factors associated with physical activity and sedentary behaviour in children and their parents throughout primary school. Briefly, data collection was conducted at three timepoints: between January 2012 and July 2013 when all participants had a child aged five to six years (Year 1, time 1), between March 2015 and July 2016 when the same child was aged eight to nine years (Year 4, time 2) and between March 2017 and May 2018 when the same child was in year 6 (aged 10–11). A total of 57 schools consented to participate at time 1 and were subsequently invited to participate at times 2 and 3. Forty-seven schools participated at time 2 and 50 participated at time 3. Across all three timepoints, data were collected from 2555 parents from 2132 families: 1195 parents were involved at time 1, 1140 at time 2, and 1233 at time 3. A total of 546 parents took part across two timepoints and 246 across three timepoints. The study received ethical approval from the University of Bristol ethics committee, and written consent was received from all participants at each phase of data collection.

### 2.2 Measures

***Characteristics.*** Parents completed a questionnaire which included information about their date of birth, gender, ethnicity, height, weight, education level, and number of children. BMI was calculated from their self-reported height and weight. Parents also reported their home postcode, and this was used to derive Indices of Multiple Deprivation (IMD scores), based upon the English Indices of Deprivation (http://data.gov.uk/dataset/index-of-multiple-deprivation). Higher scores indicate areas of higher deprivation.

***Motivation to exercise***. The Behavioural Regulation in Exercise Questionnaire (BREQ-2) was used to assess motivation to exercise (Markland & Tobin, 2004). The BREQ-2 consists of 19-items each assessing one of five forms of behavioural regulations: intrinsic (4 items e.g., ‘*I exercise because it’s fun’*), identified (4 items e.g., *‘It’*s *important to me to exercise regularly’*), introjected (3 items e.g., *‘I feel like a failure when I haven’t exercise in a while’*), external (4 items e.g., *‘I exercise because other people say I should’*), and amotivation (4 items e.g., *‘I don’t see the point in exercising’*). Due to difficulties in empirically distinguishing between identified and integrated regulation, the BREQ-2 does not assess integrated regulation (Markland & Tobin, 2004). Participants rated each item on a 5-point Likert scale ranging from 0 (*not true for me*) to 4 (*very true for me*). In the current study, the BREQ-2 subscales had good internal consistency in both the cross sectional and longitudinal samples (α > 0.7; see Table 1).

**Table 1:** Characteristics of participants and descriptive statistics of subscales in the cross-sectional regression analysis: observed and imputed data.

Characteristic			Participants at time 2 (Total *N* = 925)					Participants at time 3 (Total *N* = 891)			
			Observed data			Imputed data		Observed data			Imputed data
		*N* available	% missing	Mean (*SD*) or %	α	Mean (*SD*) or %	*N* available	% missing	Mean (*SD*) or %	α	Mean (*SD*) or %
Age (years)		925	–	41.34 (6.20)	–	41.34 (6.20)	891	–	43.32 (5.84)	–	43.32 (5.84)
Gender (% female)		925	–	72.3%	–	72.3%	891	–	72.6%	–	72.6%
BMI (kg/m^2^)		925	–	25.83 (4.84)	–	25.83 (4.84)	891	–	25.83 (4.77)	–	25.83 (4.77)
IMD score		915	.01%	14.92 (13.23)	–	14.92 (13.23)	891	–	14.44 (13.58)	–	14.44 (13.58)
Number of children		925	–	2.31 (1.07)	–	2.31 (1.07)	891	–	2.34 (0.90)	–	2.43 (0.90)
MVPA (mins/day)		925	–	50.03 (23.91)	–	50.03 (23.91)	891	–	52.28 (24.31)	–	52.28 (24.31)
Behavioural regulation	Amotivation	919	.006%	0.27 (0.56)	.81	0.27 (0.56)	885	.006%	0.23 (0.05)	.83	0.23 (0.05)
	External regulation	920	.005%	0.38 (0.60)	.78	0.38 (0.60)	883	.008%	0.31 (0.05)	.76	0.31 (0.05)
	Introjected regulation	921	.004%	1.36 (1.05)	.78	1.36 (1.05)	885	.006%	1.31 (1.06)	.79	1.31 (1.06)
	Identified regulation	921	.004%	2.63 (0.96)	.84	2.3 (0.96)	883	.008%	2.70 (.95)	.85	2.70 (0.95)
	Intrinsic regulation	921	.004%	2.51 (1.12)	.93	2.51 (1.12)	885	.006%	2.49 (1.12)	.93	2.49 (1.12)

***Physical activity.*** Participants were asked to wear a waist-worn ActiGraph wGT3X-BT accelerometer for five days, including two weekend days. Accelerometer data were processed using Kinesoft (v3.3.75; Kinesoft, Saskatchewan, Canada) in 60-s epochs. In line with recommendations for monitoring habitual physical activity in adults, analysis was restricted to participants who provided at least three days of valid data including at least 1 weekend day (Aadland & Ylvisaker, 2015; Trost, McIver, & Pate, 2005). A valid day was defined as at least 500 min of data, after excluding intervals of ≥60 min of zero counts allowing up to 2 min of interruptions. The average number of MVPA minutes per day were derived for each participant using population-specific cut points for adults (≥2020 counts per minute (Troiano et al., 2008)).

### 2.3 Data analysis

The data analysis consisted of cross-sectional analyses of time 2 and time 3 data (a cross-sectional analysis of baseline data is published elsewhere; Solomon-Moore et al., 2016) and a longitudinal analysis including data from all three timepoints. All analyses were conducted at the parent level, so where two or more parents/guardians from the same family were included in the project, each parent was treated as a separate participant. Participants were included in the cross-sectional analysis if they had valid accelerometer data, and BREQ-2 responses with not more than 1 missing item per subscale. Participants were included in the longitudinal analysis if they met the above criteria for at least one timepoint. Following recommendations for dealing with missing data, and in order to reduce bias and increase statistical power, multiple imputation using chained equations was used to impute missing data for participating parents cross-sectionally (Rubin, 1996; Tabachnick & Fidell, 2012). The imputation models included parent gender, age, ethnicity, BMI, education level, IMD score, number of children, MVPA and the five subscales of behavioural regulation. For each, 20 imputed datasets were created using 20 cycles of regression switching and estimates were combined across datasets using Rubin’s rules (Rubin, 1996).

Five independent variables, reflecting the five motivation types, were treated as continuous variables. In the cross-sectional analyses, linear regression models were used to examine associations between behavioural regulation and mean MVPA minutes per day. To identify longitudinal associations between the motivation variables and physical activity, we used a multi-level model to capture how MVPA changes over time. We also included interaction terms between behavioural regulation variables and age to explore whether change in MVPA over time differs with changes in behavioural regulation. In line with evidence for their influence on MVPA in adults (Cerin, Leslie, & Owen, 2009; Hull et al., 2010; Trost, Owen, Bauman, Sallis, & Brown, 2002), all analyses were adjusted for age, gender, number of children in the household and IMD score. In all models, robust standard errors were used to account for school clustering in the study design, and parents were clustered within families, to account for family-level similarities. All analyses were performed in Stata version 15 (StataCorp, 2017).

## 3 Results

### 3.1 Preliminary analysis

At both time 2 and time 3, missing data among participating parents was minimal, and the distributions of observed and imputed characteristics were similar (Table 1). At time 2, the sample consisted of 925 participants of whom 72% were female, with a mean age of 41.34 years (SD = 6.20), and mean BMI of 25.83 kg/m^2^ (SD = 4.84). At time 3, the sample consisted of 891 participants, of whom 73% were female, with a mean age of 43.32 years (SD = 5.84), and mean BMI of 25.83 kg/m^2^ (SD = 4.77). Mean IMD was consistent across time (14.92 at time 2 and 14.44 at time 3). Average daily MVPA increased slightly from 50.03 min (SD = 23.91) at time 2–52.28 min (SD = 24.31) at time 3. At both timepoints, means and standard deviations for all motivation variables were similar, with participants reporting higher levels of autonomous motivation than controlled motivation for exercise, with levels of identified regulation being higher than intrinsic regulation. Full descriptives for time 1 are reported elsewhere (see Solomon-Moore et al., 2016).

A total of 2374 parents had valid accelerometer data for at least one timepoint (93% of parents involved across the whole study). Of these, 463 had valid accelerometer data for two timepoints (85% of those parents involved at two timepoints) and 185 for three timepoints (75% of parents involved at three timepoints). Twenty-five parents provided no identifiable information at any timepoint and so were excluded from the analyses. Due to the study design, school attrition accounted for 244 families not taking part at time 2 and 167 families at time 3. At the family level, children moving to schools not involved in the project accounts for the drop out of 253 families. Further, as the same parent was not required to participate at every timepoint, in 227 families who had been involved at time 1, a different parent participated at time 2. A total of 302 families had a different parent participating at each timepoint. In these cases, all parents involved at any timepoint are included in the analysis.

### 3.2 Main analysis

#### 3.2.1 Cross-sectional associations between motivation and physical activity

Consistent with baseline findings (Solomon-Moore et al., 2016), fully adjusted cross-sectional regression models (Table 2) showed a positive association between identified regulation and MVPA, with a one-unit increase in identified regulation associated with a 5.4-min (95% CI [2.6, 8.3]) and 4.9 min (95% CI [1.9, 7.8]) increase in MVPA per day at time 2 and time 3, respectively. There was no evidence for an association between any other type of behavioural regulation and MVPA at time 2. At time 3, introjected regulation was negatively associated with MVPA, with a one-unit increase in introjected regulation associated with a 3.2-min decrease in MVPA per day (95% CI [-5.0, −1.4]). There was no evidence for an association between the other types of behavioural regulation and MVPA at time 3.

**Table 2:** Linear regression analyses showing associations between parent exercise motivation and their own accelerometer-estimated MVPA cross-sectionally at time 2 and time 3.

	Time 2			Time 3		
	*b*	95% CI	*p*	*b*	95% CI	
Amotivation	1.40	−1.93, 4.79	.40	−0.24	−3.97, 3.50	.90
External regulation	0.80	−2.15, 3.76	.59	0.62	−3.31, 4.55	.75
Introjected regulation	−1.49	−3.33, 0.35	.11	−3.16	−4.95, −1.39	.00
Identified regulation	5.43	2.56, 8.32	.00	4.88	1.94, 7.83	.00
Intrinsic regulation	0.83	−1.46, 3.12	.47	1.77	−0.48, 4.01	.12

**Note**. For reference, cross-sectional results from time 1 are reported in Solomon-Moore et al. (2016). Age, gender, BMI, number of children and IMD score were adjusted for in each model. Data were clustered at the school level.

#### 3.2.2 Longitudinal associations between motivation and physical activity

The full multi-level model (Model 1, Table 3) explores how parent MVPA changes across the three timepoints in relation to behavioural regulation. Daily MVPA increased by an average of 0.60 min per year (95% CI [0.17, 1.03]). Identified regulation was positively associated with MVPA, with a one-unit increase in identified regulation being associated with an average of 3.96-min more MVPA per day per year (95% CI [2.42, 5.50]).

**Table 3:** Multilevel mixed effects modelling showing associations between participant characteristics, exercise motivation and their accelerometer-estimated MVPA across all three timepoints (model 1) and including change over time variables (model 2).

	Model 1			Model 2		
	*Coef.*	95% CI	*p*	*Coef.*	95% CI	*p*
Time point	0.60	0.17, 1.03	.01	1.85	0.26, 3.44	.02
Age	−0.05	−0.22, 0.12	.56	−0.05	−0.22, 0.12	.58
Gender	−6.50	−8.59, −4.41	.00	−6.48	−8.52, −4.43	.00
BMI	−0.32	−0.53, −0.10	.00	−0.33	−0.54, −0.11	.00
Number of children	−0.55	−1.87, 0.76	.41	−0.60	−1.92, 0.72	.38
IMD	0.07	−0.01, 0.16	.10	0.07	−0.01, 0.16	.10
Amotivation	−0.88	−2.58, 0.82	.31	1.80	−6.85, 10.45	.68
External regulation	0.51	−1.28, 2.31	.58	−2.17	−8.99, 4.67	.54
Introjected regulation	−0.80	−1.68, 0.08	.08	3.75	0.70, 6.81	.02
Identified regulation	3.96	2.42, 5.50	.00	8.73	3.41, 14.05	.00
Intrinsic regulation	0.92	−0.36, 2.20	.16	−2.07	−6.54, 2.40	.36
**Change over time**						
Amotivation	–	–	–	−0.32	−1.33, 0.69	.53
External regulation	–	–	–	0.31	−0.48, 1.10	.45
Introjected regulation	–	–	–	−0.52	−0.88, −0.16	.01
Identified regulation	–	–	–	−0.55	−1.18, 0.07	.08
Intrinsic regulation	–	–	–	0.34	−0.15, 0.83	.17

**Note**. Parent age, gender, BMI, number of children and IMD score were adjusted for in each model. Data were nested in 2 levels of parent and family and analyses were clustered for school

Due to the association between time and MVPA in model 1, in model 2 we investigated whether change in MVPA over time was associated with behavioural regulation (Table 3). At time 1, identified regulation remained positively associated with MVPA with a one unit increase in identified regulation being associated with an 8.73-min increase in average daily MVPA per year (95% CI [3.41, 14.05]), however there was no association with change in MVPA over time. Introjected regulation was not associated with MVPA at time 1 but had a small negative association with change in MVPA over time, with a one unit increase in introjected regulation being associated with an average decline in daily MVPA of 0.52 min per year (95% CI [ −0.88, −0.16]).

## 4 Discussion

This study presents the first longitudinal analysis of the association between parents’ exercise motivation and accelerometer-estimated physical activity. The analyses indicate that high levels of introjected regulation can lead to a small decrease in MVPA over time. Substantiating the baseline findings from the B-Proact1v cohort, the cross-sectional analyses also show that identified regulation is consistently associated with higher levels of MVPA but was not associated with change in MVPA over time.

Our cross-sectional findings corroborate previous evidence showing that identified regulation is the type of behavioural regulation most strongly associated with physical activity behaviours in adults (Standage, Sebire, & Loney, 2008; Teixeira et al., 2012; Wilson, Sabiston, Mack, & Blanchard, 2012). These also extend the baseline findings of the B-Proact1v cohort to show that, across all three phases of the project, parents who are motivated to be physically active due to personal value engage in higher levels of MVPA than parents who are motivated in other ways. However, despite being consistently associated with higher levels of physical activity, the multi-level models suggest that identified regulation is not associated with change in physical activity over time. This is consistent with the theoretical assumption that more autonomous motivation is associated with long-term behavioural engagement (Ryan & Deci, 2017) and could indicate that identified regulation is more pertinent to behavioural maintenance, as behaviours that align with an individual’s personal values are more likely to be sustained (Kwasnicka, Dombrowski, White, & Sniehotta, 2016).

Whilst the motivational continuum proposed within SDT suggests that intrinsic motivation (i.e., enjoyment of the behaviour itself) is the strongest motivational driver of behaviour, it has been recognised in the wider literature that behaviours such as exercise or being active might be more strongly driven by what can be achieved through doing it (e.g., physical or mental health benefits, social contact with others, development of skills and feelings of competence) rather than inherent enjoyment of the activity itself (Standage & Ryan, 2012). In the present study, parents’ endorsement of intrinsic and identified types of motivation were similar, emphasising that enjoyment and satisfaction are important sources of motivation. However, the lack of association between intrinsic motivation and physical activity suggests that, for parents, enjoyment of physical activity is not sufficient to lead to action, and that personally valuing activity is a more stable motivation factor for underpinning behaviour. One explanation for this could be related to the parental role, where activities that do not directly align, and potentially compete, with core parenting duties (e.g., taking time out to be active or exercise for fun or enjoyment) are not prioritised and may even result in feelings of guilt and selfishness (Hamilton & White, 2010). The present cross-sectional findings show that when the benefits of physical activity are perceived to be personally relevant and valuable, engagement in physical activity is greater. Therefore, valuing the benefits of exercise as an individual (e.g., physical & mental health, better sleep, more energy) and/or as a parent (e.g., modelling healthy behaviours, being physically fit to “keep up” with active children; Hamilton & White, 2010) may be central to being a more physically active parent.

The cross-sectional findings showed a differential change in the association between introjected regulation and parents’ MVPA across the three timepoints, moving from a small positive association at time 1 (Solomon-Moore et al., 2016) to an increasingly negative association across times 2 and 3. The longitudinal results further highlight the impact of introjected regulation on behavioural outcomes, with a negative association between introjected regulation and change in MVPA over time. Within SDT, it is proposed that more controlled forms of motivation are detrimental to both behavioural and well-being outcomes (Ryan & Deci, 2017), yet previous longitudinal studies have found no evidence for this association (Barbeau et al., 2009; Gunnell et al., 2014) and cross-sectional research has indicated that introjected regulation may be associated with higher levels of physical activity (e.g., Brunet & Sabiston, 2011; Edmunds et al., 2006). These findings offer the first evidence of a long-term negative impact of introjected regulation on physical activity behaviour. Although further longitudinal research is warranted, given the universality of SDT we would anticipate that similar associations in the wider adult population with previous studies finding no association due to aggregating introjected and external regulations into a controlled motivation variable (e.g., Barbeau et al., 2009; Gunnell et al., 2014). However, there may also factors unique to parents that mean motivations grounded in feelings of guilt and shame have an increasing negative effect on physical activity as your child gets older. Extending previous studies, we explored the individual association of each type of behavioural regulation with change in physical activity behaviour. A potential explanation for this could be the association between introjected regulation and maladaptive social comparisons (Thogersen-Ntoumani & Ntoumanis, 2006). For example, parents may feel envy and resentment towards other parents who appear to be managing to be physically active (Hamilton & White, 2010), with such feelings having a more detrimental effect on behavioural engagement once the child is older and the parent might have more discretionary time. In terms of internal comparisons, parents of young children may accept that they cannot be physically active in the short term, hoping that they will be more active as their child gets older. If parents do not meet these self-imposed expectations when their child is older, this could result in a cyclical relationship between failure to be active and guilt. For parents these effects might be particularly salient due to the competing demands on their time.

Evidence suggests that, for adults, increasing daily MVPA by 5–10 min can have clinically meaningful health benefits (Dohrn, Kwak, Oja, Sjostrom & Hagstromer, 2018; Greaves et al., 2011). With this in mind, the data presented in this paper indicate that strategies to encourage parents to find personal value, relevance and importance in being physically active whilst ensuring that feelings of guilt or shame are not induced, may be particularly important for promoting increased physical activity engagement with the potential for meaningful impact on health outcomes. This requires environments that support rather than thwart the three psychological needs of autonomy, competence and relatedness (Ryan & Deci, 2017). Such environments are characterised by the provision of choice (e.g., when and how to be active/exercise), optimal challenge (e.g., one’s exercise goals/plans are neither too easy nor too hard to achieve), and strong connections with others (e.g., being active is normal/accepted amongst one’s friends, family, or colleagues). Additionally, and particularly relevant for the promotion of identified regulation, may be the provision of a personally relevant and valuable rationale for being active, which has been shown to promote long-term behavioural engagement (Samdal, Eide, Barth, Williams, & Meland, 2017). Qualitative evidence suggests that long-term goals can be abstract and undermine physical activity and, as such, physical activity messages should emphasise that being active can also be immediately gratifying and help people cope with or manage their daily goals (Segar, Taber, Patrick, Thai, & Oh, 2017). Research on the content of exercise goals (essentially benefits of exercise) shows that goals such as health, social affiliation and development of exercise competence or skills are associated with greater autonomous motivation and psychological well-being (Sebire, Standage & Vansteenkiste, 2009). For parents, effective messages could include promoting physical activity as an important family activity that provides the opportunity to spend time together and interact with others, increase energy levels, and to relax and escape daily pressures (Segar et al., 2017). However, further qualitative work is required to inform the development of specific health messaging that aligns with identified regulation.

## 5 Limitations and future directions

The strengths of the study lie in the theoretically-grounded approach to motivation, longitudinal data collected from the same cohort on three occasions over a five-year period and the use of accelerometers to estimate levels of MVPA which when combined have made a novel contribution to the literature. However, limitations should be acknowledged. First, the sample was largely female, and therefore is more representative of mothers than parents in general. However, given the universality of SDT, and supporting evidence indicating no gender differences in behavioural regulation as measured through BREQ-2, we would not anticipate significant differences in findings in a more male-dominated sample (Guerin, Bales, Sweet, & Fortier, 2012). Additionally, participating parents were generally more active than average in the UK (Craig, Mindell, & Hirani, 2011) and had high levels of self-determined motivation. This may limit the generalisability of the findings to the wider parent population and may also have limited the extent to which we can observe change over time. Whilst we did not have the power in the present study, in future researchers may want to look at the differences in motivation between parents with low physical activity levels, and those with high physical activity levels. A further limitation is that the BREQ-2 questionnaire used to measure motivation does not assess integrated regulation which sits on the SDT continuum of motivation between identified and intrinsic motivation types and represents the assimilation of the behaviour with one’s values, goals, and sense of self (e.g., “I am active”; Ryan & Deci, 2017).

## 6 Conclusion

This study is the first to assess the longitudinal associations between parent’s behavioural regulation to exercise in relation to accelerometer-estimated physical activity. The results indicate that motivation grounded in the personal meaning and value of exercise is associated with higher levels of MVPA. Additionally, the results suggest that being motivated by feelings of guilt and shame impact negatively on MVPA over time. Therefore, interventions that promote greater enjoyment, personal relevance and value, whilst also ensuring that guilt is not promoted, may offer promise for the facilitation of greater long-term physical activity engagement in parents.

## Conflicts of interest

We have no competing interests to report.

## Funding

This work was funded by the British Heart Foundation (Grant numbers: PG/11/51/28986 and SP 14/4/31123).
